# Preoperative dynamic breast magnetic resonance imaging kinetic features using computer-aided diagnosis: Association with survival outcome and tumor aggressiveness in patients with invasive breast cancer

**DOI:** 10.1371/journal.pone.0195756

**Published:** 2018-04-12

**Authors:** Sang Yu Nam, Eun Sook Ko, Yaeji Lim, Boo-Kyung Han, Eun Young Ko, Ji Soo Choi, Jeong Eon Lee

**Affiliations:** 1 Department of Radiology, Gachon University Gil Medical Center, Gachon University of Medicine and Science, Incheon, Korea; 2 Department of Radiology, Samsung Medical Center, Sungkyunkwan University School of Medicine, 81 Irwon-ro, Gangnam-gu, Seoul, Korea; 3 Department of Applied Statistics, Chung-Ang University, 84 Heukseok-ro, Dongjak-gu, Seoul, Korea; 4 Department of Surgery, Samsung Medical Center, Sungkyunkwan University School of Medicine, 81 Irwon-ro, Gangnam-gu, Seoul, Korea; Taipei Medical University, TAIWAN

## Abstract

**Objectives:**

To evaluate whether preoperative breast dynamic contrast-enhanced (DCE) magnetic resonance (MR) imaging kinetic features, assessed using computer-aided diagnosis (CAD), can predict survival outcome and tumor aggressiveness in patients with invasive breast cancer.

**Materials and methods:**

Between March and December 2011, 301 women who underwent preoperative DCE MR imaging for invasive breast cancer, with CAD data, were identified. All MR images were retrospectively evaluated using a commercially available CAD system. The following kinetic parameters were prospectively recorded for each lesion: initial peak enhancement, the proportion of early phase medium and rapid enhancement, and the proportion of delayed phase persistent, plateau, and washout enhancement. The Cox proportional hazards model was used to determine the association between the kinetic features assessed by CAD and disease-free survival (DFS). The peak signal intensity and kinetic enhancement profiles were compared with the clinical-pathological variables.

**Results:**

There were 32 recurrences during a mean follow-up time of 55.2 months (range, 5–72 months). Multivariate analysis revealed that a higher peak enhancement (DFS hazard ratio, 1.004 [95% confidence interval (CI): 1.001, 1.006]; *P =* .013) on DCE MR imaging and a triple-negative subtype (DFS hazard ratio, 21.060 [95% CI: 2.675, 165.780]; *P* = .004) were associated with a poorer DFS. Higher peak enhancement was significantly associated with a higher tumor stage, clinical stage, and histologic grade.

**Conclusions:**

Patients with breast cancer who showed higher CAD-derived peak enhancement on breast MR imaging had worse DFS. Peak enhancement and volumetric analysis of kinetic patterns were useful for predicting tumor aggressiveness.

## Introduction

Dynamic contrast-enhanced (DCE) magnetic resonance (MR) imaging is the most sensitive modality for breast cancer detection, and is commonly used for the preoperative evaluation of newly diagnosed breast cancer cases [1–3]. Morphological features and patterns of contrast enhancement on MR imaging provide important information, not only of tumor histological features, such as vascularization, but also survival outcome prediction [4–6].

Regarding evaluating kinetic features, there are several methods to assess the kinetic enhancement patterns of breast tumor obtained from DCE MR imaging [7]. For quantitative analysis, pharmacokinetic models, such as Tofts and extended Tofts models, are standard for analyzing DCE MRI data. These models are suitable when the tissue under investigation is weakly vascularized or in the presence of a negligible intravascular concentration of contrast agent [8, 9]. The Tofts model considers intravascular space as one compartment and extracellular extravascular space as the other compartment, excluding the functionality of intravascular space. Computation of pharmacokinetic parameters requires curve fitting to concentration–time curves for multiple voxels. This is typically time-consuming and hinders online access of pharmacokinetic maps by radiologists [10]. Most commonly, breast kinetic enhancement patterns are measured semi-quantitatively using modest temporal resolution with at least two to three post-contrast T1-weighted acquisitions, with k-space centered at approximately 90–120 seconds after contrast injection for the first post-contrast images [7]. Using the data obtained at each of these time points, a time-signal intensity curve can be determined for a given lesion or region of interest. Despite its high utility in functional analysis, kinetic assessment using a manually drawn region of interest (ROI) is limited because it only reflects partial pixel information of the lesion. Moreover, ROI placement and interpretation are operator-dependent and time-consuming.

Despite the controversy of whether it improves the diagnostic performance of MR imaging, computer-aided diagnosis (CAD) systems have the advantage of being easy to use, and are widely clinically available to automatically assess kinetic information and perform volumetric analysis for breast lesions [11–14]. CAD systems eliminate the need for labor-intensive tasks, such as manual drawing of ROI by a technician or radiologist. In addition, CAD has the potential to increase observer reproducibility in DCE MR imaging by performing automatic and quantitative analysis of the contrast material uptake [15–17].

The correlation between kinetic parameters on CAD and the histological characteristics of breast cancer have been evaluated [18]. CAD-derived kinetic information may be used to assess tumor aggressiveness, including hormone receptor status and histologic grade. In terms of survival outcomes, Yi et al. [19] reported that a smaller reduction in the washout component on DCE MR imaging assessed using CAD after neoadjuvant chemotherapy (NAC) in breast cancer patients was independently associated with a worse recurrence-free survival (RFS) and overall survival [19]. In a preoperative setting, Dietzel et al. [20] evaluated the potential of DCE MR imaging to predict disease-related death using CAD on 115 patients, finding that total tumor volume, initial peak enhancement, and time to peak enhancement showed a negative correlation with survival. The persistent curve type, however, showed a positive correlation with survival. Baltzer et al. [21] analyzed the enhancement kinetics of 59 patients using CAD and found that a washout component could be identified as a significant and independent predictor of distant metastasis occurrence. However, because their studies only included a small sample size, patients in NAC setting, or a qualitative assessment using in-house software, there is currently a lack of reports evaluating the correlation between preoperative MR imaging kinetic parameters assessed using a commercially available CAD with recurrence outcomes in patients with breast cancer.

Therefore, the purpose of our study was to evaluate whether the kinetic features of preoperative MRI, assessed using CAD, can predict survival outcomes in patients with newly diagnosed invasive breast cancer.

## Materials and methods

### Patients

This retrospective study was approved by the Institutional Review Board of the Samsung Medical Center (SMC IRB 2017-03-057) and waived the need for informed patient consent.

Between March and December 2011, 823 consecutive women (mean age of 50 years, range 24–85 years) who had undergone surgery for invasive breast cancer were identified. The inclusion criteria for our study were as follows: (a) completion of preoperative DCE MR imaging with CAD at our institution, (b) initial unilateral breast biopsy-proven malignancy with a final pathologic diagnosis of invasive breast carcinoma, (c) a lesion manifesting as a mass on MR imaging. We identified 753 invasive breast carcinomas that met these criteria. We excluded cases where MR imaging was performed after diagnosis by vacuum-assisted or excisional biopsy (n = 97). Patients treated with NAC (n = 81), those who underwent breast MR imaging with a 3-T scanner (n = 264), and those who had a follow-up period of less than 3 months (n = 10) were also excluded. To exclude any magnetic strength effects during the MR examination, we only included patients who underwent 1.5-T MRI, ensuring that the MR images were obtained under homogeneous conditions. Finally, 301 women, aged 26–80 years (mean age 51 years) were included in our study. S1 Dataset provides detailed characteristics of patients (online). The mean interval between preoperative MR examination and surgery was 13 days (range 1–37 days).

### MR imaging protocol

MR imaging was performed using a 1.5-T system (Achieva; Philips Medical Systems, Best, The Netherlands) with a dedicated bilateral phased-array breast coil, with the patient in a prone position. The MR imaging examination consisted of turbo spin-echo T1- and T2-weighted sequences, and a three-dimensional DCE sequence. The DCE MR imaging sequences were performed using the following parameters: repetition time (TR) = 6.5 ms, echo time (TE) = 2.5 ms, slice thickness = 1.5 mm, flip angle = 10°, matrix size = 376 × 374 mm, field of view (FOV) = 32 × 32 cm. Axial DCE MR imaging was performed using 1 precontrast and 6 postcontrast dynamic series. Contrast-enhanced images were acquired 30, 90, 150, 210, 270, and 330 seconds after the contrast material injection. The length of each dynamic series was 60.05 seconds. A 0.1 mmol/kg bolus of gadobutrol (Gadovist; Bayer Healthcare Pharmaceutical, Berlin, Germany) was injected for dynamic contrast imaging, before a 20-mL saline flush.

### Assessment of MR imaging and computer-aided diagnosis

The DCE MR images were transferred to a CAD system (CADstream, version 4.1.3, Merge Healthcare, Chicago, IL, USA) that analyzed the signal intensities within each voxel of the FOV obtained during the dynamic sequences. The American College of Radiology Breast Imaging Reporting and Data System (ACR BI-RADS) MR lexicon [22] defined a persistent kinetic pattern as a continuous increase in signal over time. A plateau is defined as a signal intensity that does not change over time, after an initial rise. When the signal intensity decreases by more than 10%, after its highest point following its initial rise, it is defined as a washout. Using CAD stream, a color overlay showing the changes in signal intensity over time was automatically generated in all slices using a predefined minimum threshold. We defined the minimum threshold as 50%, or a greater increase in pixel-by-pixel comparison between the precontrast and second post-contrast images. When the relative enhancement increase was more than 100% of the precontrast image, it was classified as “rapid uptake,” and when it was 50–100% it was classified as “medium uptake.” When the signal intensity continued to show an increase of more than 10% in the sixth postcontrast-enhanced images, compared to the second postcontrast-enhanced images, it was classified as “persistent.” When the signal intensity showed a decrease of more than 10%, it was classified as a “washout,” and when the signal intensity continued to show within a 10% range, it was classified as a “plateau.” We maintained a single threshold value for early and delayed phase enhancements across all patients. Volumetric assessment of the kinetic components refers to the assessment of the percentage volume of each kinetic component found within the tumors at both early and delayed phases of enhancement. The CAD reports included the following kinetic features: the initial peak enhancement values, proportions of early phase medium and rapid enhancements, and the proportion of delayed phase persistent, plateau, and washout enhancements. The following three kinetic curve patterns presented by CAD were also included in the CAD reports: type 1, delayed persistent enhancement pattern; type 2, delayed plateau enhancement pattern; and type 3, delayed washout enhancement pattern. The CAD reports were stored prospectively by interpreting radiologists, and square ROI of the entire tumor was automatically segmented when an operator chose the image slice showing the largest diameter. For cases that had multiple cancers on the MRI, we used the values of the index cancer.

The MR imaging findings were retrospectively evaluated according to BI-RADS MR lexicon [22] by two board-certified radiologists (S.Y.N., E.S.K., with 6 and 10 years of experience in breast MR imaging, respectively, in consensus. In cases with discrepancy in interpretation between the two readers, a third radiologist (J.S.C. with 7 years of experience in breast MR imaging) reviewed the images to reach a consensus. The radiologists assessed the shape (oval, round, or irregular), margin (circumscribed, irregular, or spiculated), and internal enhancement characteristics (homogeneous, heterogeneous, rim, or dark internal septation) of each mass.

### Histopathological features

The following parameters of the pathological reports of either breast-conserving surgery or mastectomy specimens were reviewed: pathologic diagnosis, histologic grade, surgical margin status, presence of an extensive intraductal component (EIC), lymphovascular invasion (LVI), estrogen receptor (ER), progesterone receptor (PR), human epidermal growth factor receptor 2 (HER2), and Ki-67 expression status. Positivity for ER and PR was defined using a cut-off value of greater than 1% positively stained nuclei. Immunohistochemical HER2 scores of 3+ (strong homogeneous staining) were considered positive. In cases of HER2 scores of 2+, HER2 gene amplification was confirmed using silver in situ hybridization (SISH). For Ki-67 expression status, nuclear staining of at least 14% was considered to indicate a high level of expression. Breast cancers were divided into three molecular subtypes based on the immunohistochemical or SISH findings for ER, PR, HER2 as follows: luminal (hormone receptor positive and any of HER2 status), HER2-enriched (hormone receptor negative and HER2 positive), and triple-negative (hormonal receptor negative and HER2 negative) [23]. Tumors were divided into three histological groups: invasive ductal carcinoma, invasive lobular carcinoma, and others. The surgical margin status was classified as positive or close (<2 mm), or negative. Because frozen biopsy was routinely performed during surgery at our institution, immediate repeat excision was performed on the same day for patients showing a positive margin in the frozen biopsy. Only the final results of the surgical margin were analyzed. We reviewed the patients’ medical records to identify whether they received adjuvant chemotherapy, adjuvant radiation therapy, or adjuvant endocrine therapy. We also reviewed the TNM stage after surgery and the follow-up information from the medical records including imaging studies for each patient.

### Statistical analyses

Breast cancer recurrence was defined as local recurrence (limited to the ipsilateral breast or chest wall), regional recurrence (ipsilateral axillary, infraclavicular, or supraclavicular lymph nodes), contralateral breast, or distant metastasis to other parts of the body. DFS and disease-specific survival (DSS) were calculated from the date of surgery to that of breast cancer recurrence, the date of death, the date last known to have no evidence of disease, or the date of the most recent follow-up. Patients without an event were censored at the date of the most recent follow-up, regardless of whether they were scheduled for future follow-up or they had been lost to follow-up.

The Cox proportional hazards model was used to determine the association between MR imaging kinetic features derived from CAD and DFS or DSS. The peak signal intensity and volumetric assessment of different kinetic components were compared to the clinical-pathological variables using a Student’s *t-*test and analysis of variance (ANOVA).

Statistical significance was set at *P* < .05. Variables with *P* < .05 on the univariate analysis were entered as the input variables for a multivariate model. All statistical analyses were performed by a dedicated statistician using R statistical software (version 3.2.4; R Foundation for Statistical Computing, Vienna, Austria).

## Results

### Patients characteristics and survival outcome

The mean follow-up time was 55.2 months (range, 5–72 months). There were 32 recurrences in 32 patients (7 local, 6 regional, 5 contralateral breasts, 14 distant) at a mean of 33.3 months (range, 5–72 months).

Of the 301 study lesions, 272 (90.4%) were invasive ductal carcinomas, 11 (3.7%) were invasive lobular carcinomas, and 18 (5.9%) were other types of carcinoma. Of the 18 other carcinomas, 5 were mucinous, 3 were mixed invasive ductal and mucinous, 3 were invasive apocrine, 3 were invasive micropapillary, 2 were medullary, 1 was adenoid cystic, and 1 was tubular. Among 301 patients, 59 (19.6%) patients underwent mastectomy and 242 (80.4%) patients received breast-conserving surgery. For resection margin, most were negative (80.1%) while 19.9% were close (<2mm) or positive.

### Survival analysis: Univariate and multivariate

**Fig 1:**
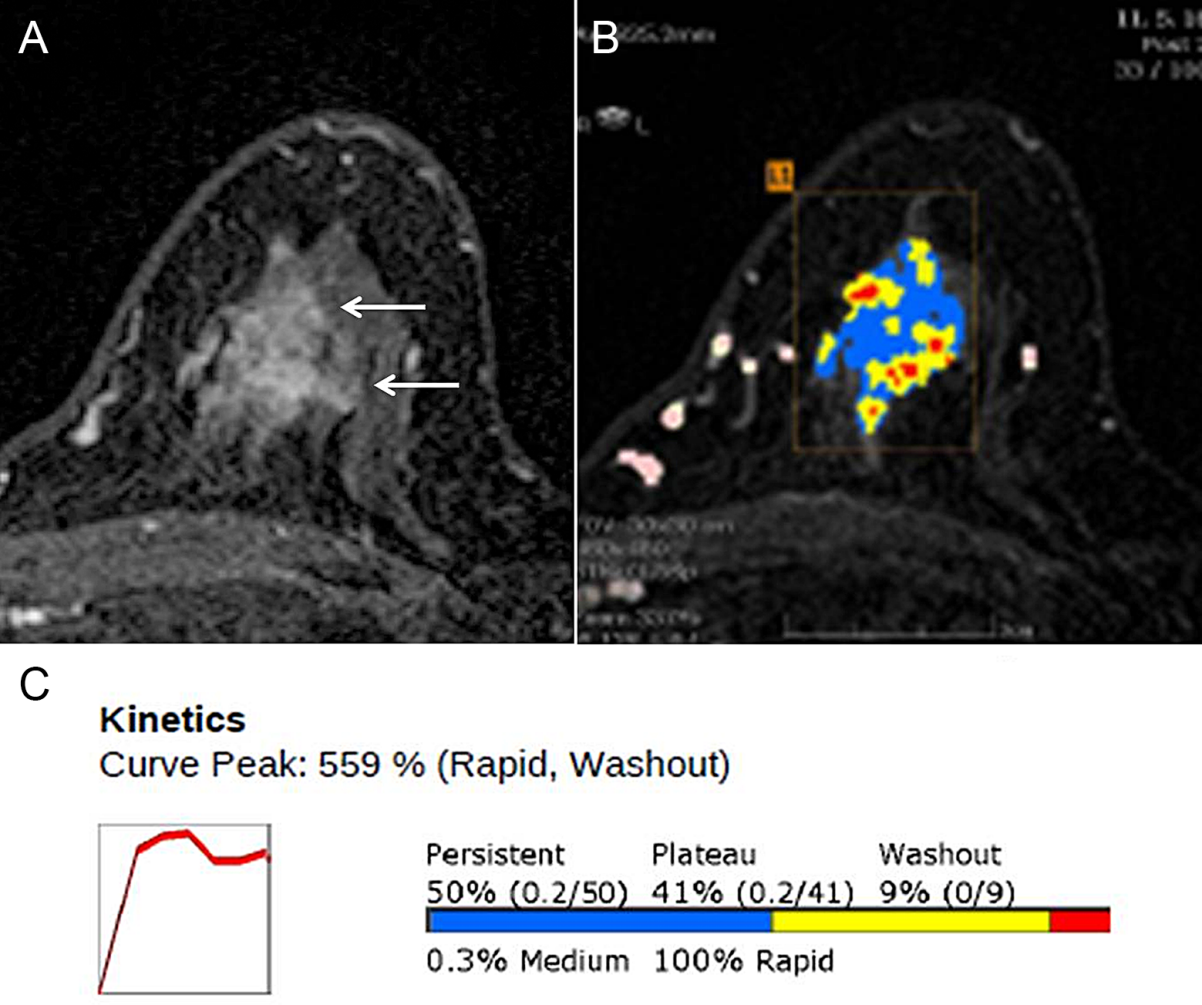
Magnetic resonance (MR) images with computer-aided detection (CAD) Color Overlay Map in a 66-year-old Woman with grade 2 invasive ductal carcinoma in the left breast. (**A**) Axial contrast-enhanced T1-weighted MR image shows a 26-mm irregular mass (arrows). (**B**) Axial maximum-intensity-projection MR image shows CAD color overlay over the breast mass. L1 denotes the first lesion of the left breast. Areas in red, yellow, and blue indicate a rapid washout-type delayed enhancement, plateau-type delayed enhancement, and persistent-type delayed enhancement pattern, respectively. **(C)** Kinetic curve graph showing rapid initial enhancement and rapid washout-type curve. The initial peak enhancement value was 559%. With respect to the delayed phase enhancement, 9% of the mass showed washout, 50% of the mass showed a persistent-type curve, and 41% showed a plateau-type curve. Regional recurrence was diagnosed in the ipsilateral axillary lymph node 26 months after surgery.

**Fig 2:**
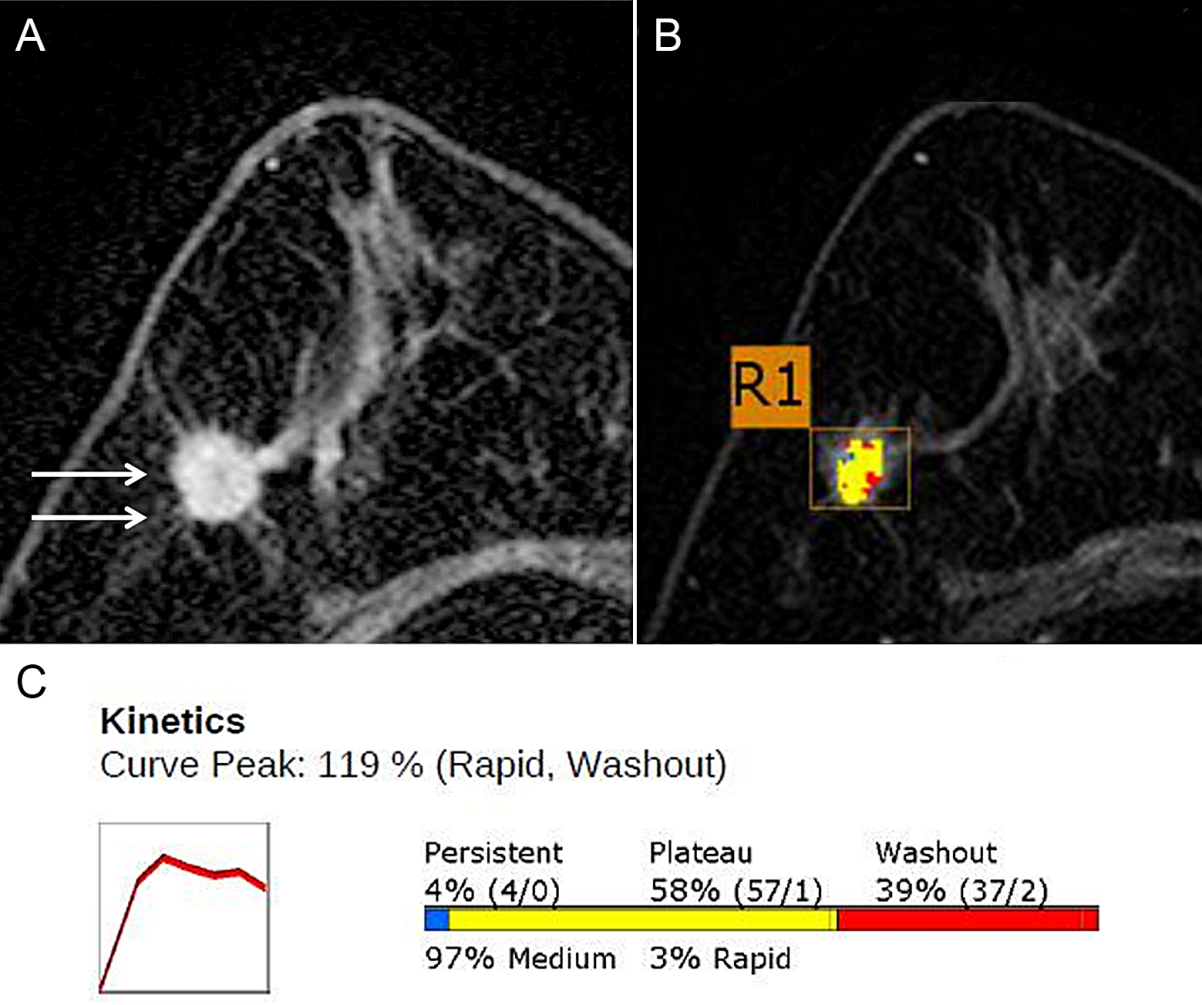
Magnetic resonance (MR) Images with Computer-aided detection (CAD) Color Overlay Map in a 71-year-old Woman with grade 2 invasive ductal carcinoma in the right breast. (**A**) Axial contrast-enhanced T1-weighted MR image shows a 15-mm irregular mass (arrows). (**B**) Axial maximum-intensity-projection MR image shows a CAD color overlay over the breast mass. R1 denotes the first lesion of the right breast. Areas in red, yellow, and blue indicate rapid washout-type delayed enhancement, plateau-type delayed enhancement, and persistent-type delayed enhancement patterns, respectively. **(C)** Graph of the kinetic curve shows rapid initial enhancement and rapid washout-type curve. The initial peak enhancement value was 119%. With respect to the delayed phase enhancement, 39% of the mass showed washout, 4% of the mass showed a persistent-type curve, and 58% showed a plateau-type curve. There was no recurrence during the 65-month follow-up period.

Univariate analysis of clinicopathological variables associated with DFS is presented in Table 1. Among the clinical-pathological variables, T2 stage (DFS hazard ratio, 2.053; *P* = .049), N3 stage (DFS hazard ratio, 4.836; *P* = .005), histologic grade 2 (DFS hazard ratio, 4.490; *P* = .048), histologic grade 3 (DFS hazard ratio, 9.782; *P* = .002), ER negativity (DFS hazard ratio, 5.243; *P* < .0001), PR negativity (DFS hazard ratio, 4.402; *P* = .0001), triple-negative subtype (DFS hazard ratio, 7.093; *P* < .0001), and not receiving adjuvant endocrine therapy (DFS hazard ratio, 3.968; *P* = .0001) were associated with worse DFS outcomes. With regard to the CAD-derived kinetic features, the mean peak enhancement value of the recurrence group was higher than that of the non-recurrence group (310.19% [median, 305%; interquartile range, 236–384.8%] vs 252.13% [median, 235%; interquartile range, 191–297%], respectively) and the higher peak signal enhancement (DFS hazard ratio, 1.004; *P* = .001) was significantly associated with a worse DFS on univariate analysis (Figs 1 and 2; Table 2). Although the mean value of the washout component was higher in the recurrence group than non-recurrence group (39.19% [median, 40.5%; interquartile range, 24.50–54.25%] vs 38.08% [median, 34%; interquartile range, 15–60%], respectively), there was no statistically significant difference in DFS between the two groups (DFS hazard ratio, 1.001; *P* = .834). The three kinetic curve patterns presented by CAD were not significantly difference in DFS between the two groups (*P* > .05).

**Table 1:** Univariate Cox proportional hazard analysis of clinicopathological variables with survival outcomes.

Variables	No. of patients (recurrence)		Hazard ratio	95% CI	*P* value
	No (N = 269)	Yes (N = 32)			
Mean age (years)	51.2 (28–80)	49.5 (26–76)	0.988	0.954, 1.023	0.497
T stage					
1	169 (62.8%)	14 (43.8%)	Reference		
2	90 (33.5%)	16 (50.0%)	2.053	1.002, 4.207	0.049
3	9 (3.3%)	2 (6.3%)	2.777	0.631, 12.223	0.177
4	1 (0.4%)	0 (0%)	0	0.000, ∞	0.997
N stage					
0	170 (63.2%)	17 (53.1%)	Reference		
1	77 (28.6%)	9 (28.1%)	1.153	0.514, 2.586	0.731
2	16 (5.9%)	2 (6.3%)	1.258	0.291, 5.444	0.759
3	6 (2.2%)	4 (12.5%)	4.836	1.626, 14.386	0.005
Stage					
1	126 (46.8)	10 (31.3%)	Reference		
2	115 (42.8%)	16 (50.0%)	1.667	0.756, 3.673	0.205
3	28 (10.4%)	6 (18.8%)	2.580	0.938, 7.100	0.066
Pathologic type					
IDC	242 (90.0%)	30 (93.8%)	Reference		
ILC	10 (3.7%)	1 (3.1%)	0.850	0.116, 6.233	0.873
Others	17 (6.3%)	1 (3.1%)	0.663	0.090, 4.862	0.686
Histologic grade					
1	88 (32.7%)	2 (6.3%)	Reference		
2	114 (42.4%)	13 (40.6%)	4.490	1.013, 19.896	0.048
3	67 (24.9%)	17 (53.1%)	9.782	2.260, 42.342	0.002
ER					
Positive	202 (75.1%)	11 (34.4%)	Reference		
Negative	67 (24.9%)	21 (65.6%)	5.243	2.527, 10.880	<0.0001
PR					
Positive	185 (68.8%)	10 (31.2%)	Reference		
Negative	84 (31.2%)	22 (68.8%)	4.402	2.084, 9.299	0.0001
HER2					
Positive	59 (21.9%)	5 (15.6%)	Reference		
Negative	210 (78.1%)	27 (84.4%)	1.511	0.582, 3.923	0.397
p53					
Positive	91 (33.8%)	14 (43.8%)	Reference		
Negative	178 (66.2%)	18 (56.3%)	0.675	0.336, 1.357	0.270
Molecular subtype					
Luminal	203 (75.5%)	12 (37.5%)	Reference		
HER2-enriched	30 (11.2%)	2 (6.3%)	1.160	0.260, 5.182	0.846
Triple-negative	36 (13.4%)	18 (56.3%)	7.093	3.412, 14.745	<0.0001
Ki-67					
>14%	164 (61.0%)	25 (78.1%)	Reference		
≤14%	105 (39.0%)	7 (21.9%)	0.460	0.199, 1.063	0.069
LVI					
Present	86 (32.0%)	14(43.8%)	Reference		
Absent	183 (68.0%)	18 (56.3%)	0.609	0.303, 1.225	0.164
EIC					
Present	54 (20.1%)	7 (21.9%)	Reference		
Absent	215 (79.9%)	25 (78.1%)	0.960	0.416, 2.220	0.924
Operation method					
Breast-conserving surgery	218 (81.0%)	24 (75.0%)	Reference		
Mastectomy	51 (19.0%)	8 (25.0%)	1.368	0.614, 3.045	0.443
Resection margin					
Negative	215 (79.9%)	26 (81.3%)	Reference		
Close (<2 mm) or positive	54 (20.1%)	6 (18.8%)	0.901	0.371, 2.189	0.817
Adjuvant chemotherapy					
No	74 (27.5%)	4 (12.5%)	Reference		
Yes	195 (72.5%)	28 (87.5%)	0.412	0.145, 1.175	0.097
Adjuvant radiation therapy					
No	38 (14.1%)	6 (18.8%)	Reference		
Yes	231 (85.9%)	26 (81.2%)	1.462	0.601, 3.559	0.403
Adjuvant endocrine therapy					
No	70 (26.0%)	19 (59.4%)	Reference		
Yes	199 (74.0%)	13 (40.6%)	3.968	1.961, 8.065	0.0001

CI = confidence interval, IDC = invasive ductal carcinoma, ILC = invasive lobular carcinoma, ER = estrogen receptor, PR = progesterone receptor, HER2 = human epidermal growth factor receptor 2, LVI = Lymphovascular invasion, EIC = Extensive intraductal component

**Table 2:** Univariate Cox proportional hazard analysis of morphologic and kinetic features on MR imaging with survival outcomes.

Variables	No. of patients (recurrence)		Hazard ratio	95% CI	*P* value
	No (n = 269)	Yes (n = 32)			
Mass shape					
Oval	40 (14.9%)	5 (15.6%)	Reference		
Round	27 (10.0%)	3 (9.4%)	0.926	0.221, 3.875	0.916
Irregular	202 (75.1%)	24 (75.0%)	1.020	0.389, 2.673	0.968
Mass margin					
Circumscribed	25 (9.3%)	3 (9.4%)	Reference		
Not circumscribed	244 (90.7%)	29 (90.6%)	1.072	0.327, 3.520	0.909
Mass internal enhancement					
Homogeneous	32 (11.9%)	2 (6.2%)	Reference		
Heterogeneous	204 (75.8%)	22 (68.8%)	1.829	0.430, 7.779	0.414
Rim enhancement	33 (123%)	8 (25.0%)	4.004	0.850, 18.858	0.079
Peak signal intensity (%)*	252.13 (77–986)	310.19(132–559)	1.004	1.002, 1.006	0.001
Early phase enhancement §					
Medium (%)	7.96	4.00	0.980	0.948, 1.013	0.234
Rapid (%)	92.08	96.03	1.020	0.987, 1.054	0.236
Delayed phase enhancement §					
Persistent (%)	21.22	19.3	0.997	0.982, 1.013	0.732
Plateau (%)	40.69	41.56	1.001	0.983, 1.021	0.886
Washout (%)	38.08	39.19	1.001	0.989, 1.014	0.834
Kinetic curve presented by CAD					
Type 1 (delayed persistent enhancement pattern)	2 (0.7%)	0 (0%)	Reference		
Type 2 (delayed plateau enhancement pattern)	13 (4.8%)	2 (6.3%)	4072785.2191	0, Inf	0.997
Type 3 (delayed washout enhancement pattern)	254 (94.4%)	30 (93.7%)	3274787.6777	0, Inf	0.997

* Numbers represent the mean values with ranges

§ Numbers represent percentages

CI = confidence interval

Multivariate analysis revealed that higher peak enhancement (DFS hazard ratio, 1.004 [95% confidence interval [CI]: 1.001, 1.006]; *P =* .013) and triple-negative subtype (DFS hazard ratio, 21.060 [95% CI: 2.675, 165.780]; *P* = .004) were independently associated with worse DFS outcomes (Table 3). Survival analysis for DSS was not performed because there were only six deaths in our study population [24].

**Table 3:** Multivariate Cox proportional hazard analysis of clinicopathological variables and MR imaging kinetic parameters with survival outcomes.

Variable	Disease-free survival		
	*P value*	Hazard ratio	95% CI
Peak enhancement	0.013	1.004	1.001, 1.006
T stage			
1		Reference	
2	0.220	1.669	0.737, 3.779
3	0.215	3.046	0.524, 17.698
4	0.998	0.000	0.000, ∞
N stage			
0		Reference	
1	0.591	1.267	0.534, 3.004
2	0.836	0.851	0.185, 3.914
3	0.081	3.040	0.872, 10.598
Histologic grade			
1		Reference	
2	0.131	3.227	0.704, 14.784
3	0.176	3.162	0.596, 16.775
Molecular subtype			
Luminal		Reference	
HER2-enriched	0.306	3.538	0.314, 39.828
Triple-negative	0.004	21.060	2.675, 165.780
Adjuvant endocrine therapy			
No	0.263	3.282	0.409, 26.305
Yes		Reference	

CI = confidence interval, HER2 = human epidermal growth factor receptor 2

### Correlation between MR kinetic parameters and clinical-pathological variables

The correlation between CAD-derived MR kinetic features with clinicopathological variables are presented in Table 4. Higher peak enhancement was significantly associated with a higher T stage, higher clinical stage, and a higher histologic grade. Pathologic diagnosis of invasive ductal carcinoma, higher histologic grade, and triple-negative subtype were significantly associated with a higher washout component on the delayed enhancement phase on DCE MR imaging. Higher histologic grade, ER negativity, PR negativity, and p53 positivity were associated with a higher mean percentage volume of rapid enhancement components on early phase enhancement. On the contrary, lower histologic grade, ER positivity, PR positivity, and p53 negativity were associated with a higher mean percentage volume of medium enhancement components on early phase enhancement. Persistent enhancement on delayed phase was seen in tumors with a lower histologic grade.

**Table 4:** Relationship between MR imaging kinetic features and various clinicopathological findings.

Variable	Peak signal intensity		Early enhancement phase				Delayed enhancement phase					
	Value (%)	*P*	Medium (%)	*P*	Rapid (%)	*P*	Persistent (%)	*P*	Plateau (%)	*P*	Washout (%)	*P*
T stage		<0.0001		0.865		0.865		0.615		0.149		0.611
1	235.634		8.139		91.896		21.980		40.783		37.208	
2	293.528		6.680		93.359		20.141		39.509		40.371	
3	297.818		6.418		93.636		15.273		52.727		32.182	
4	239.000		0.000		100.000		1.000		45.000		54	
N stage		0.097		0.971		0.971		0.373		0.950		0.412
0	248.460		7.264		92.775		21.979		40.481		37.530	
1	278.151		7.687		92.337		17.514		40.921		41.584	
2	245.833		9.171		90.889		25.500		41.667		32.889	
3	294.200		8.360		91.700		25.100		43.700		31.000	
Stage		0.0003		0.797		0.801		0.620		0.615		0.366
1	232.765		7.512		92.529		21.882		40.596		37.508	
2	283.611		7.097		92.931		19.560		40.238		40.216	
3	262.971		9.317		90.7353		23.1765		43.6471		33.1471	
Pathologic type		0.585		0.401		0.407		0.054		0.485		0.015
IDC	259.768		7.106		92.927		19.950		40.427		39.615	
ILC	262.273		10.706		89.455		32.636		46.727		20.473	
Others	233.778		12.087		87.944		30.056		42.556		27.556	
Histologic grade		0.032		0.029		0.030		0.019		0.299		0.006
1	234.644		8.908		91.144		26.713		41.589		31.701	
2	266.126		9.327		90.709		19.527		41.939		38.552	
3	271.833		3.356		96.667		17.169		38.179		44.610	
ER		0.661		0.039		0.040		0.247		0.181		0.053
Positive	256.612		8.841		91.202		22.035		41.686		36.259	
Negative	262.386		4.376		95.648		18.555		38.602		42.878	
PR		0.505		0.038		0.040		0.344		0.273		0.110
Positive	255.364		9.035		91.010		21.972		41.632		36.362	
Negative	263.717		4.776		95.245		19.262		39.226		41.564	
HER2		0.085		0.401		0.406		0.560		0.206		0.189
Positive	278.141		5.944		94.078		19.636		38.234		42.126	
Negative	252.949		7.965		92.076		21.391		41.473		37.132	
p53		0.376		0.026		0.027		0.064		0.128		0.550
Positive	265.543		4.550		95.476		17.568		42.962		39.467	
Negative	254.429		9.134		90.908		22.866		39.618		37.512	
Molecular subtype		0.765		0.096		0.098		0.210		0.149		0.016
Luminal	255.586		8.879		91.163		22.133		41.903		35.945	
HER2 enriched	267.188		4.025		96.000		14.225		35.500		50.284	
Triple-negative	263.870		4.264		95.759		20.604		39.463		39.985	
Ki-67		0.177		0.086		0.087		0.103		0.955		0.163
>14%	264.524		6.236		93.799		19.301		40.831		39.866	
≤14%	247.813		9.72		90.313		23.914		40.707		35.373	
LVI		0.143		0.056		0.057		0.167		0.023		0.747
Present	270.710		4.875		95.160		18.341		44.150		37.482	
Absent	252.134		8.859		91.179		22.349		39.110		38.548	
EIC		0.056		0.759		0.760		0.851		0.021		0.081
Present	251.295		6.934		93.099		21.528		45.574		32.820	
Absent	260.088		7.688		92.350		20.888		39.568		39.560	
Operation method		0.055		0.069		0.069		0.640		0.339		0.303
Breast-conserving surgery	252.649		6.653		93.384		20.702		40.290		38.984	
Mastectomy	281.509		11.152		88.881		22.312		42.814		34.953	
Mass shape		0.056		0.856		0.855		0.822		0.066		0.188
Oval	247.111		7.557		92.467		19.409		35.978		44.667	
Round	219.933		9.169		90.867		22.870		37.540		39.567	
Irregular	265.628		7.314		92.726		21.092		42.173		36.723	
Mass margin		0.569		0.981		0.972		0.623		0.802		0.795
Circumscribed	247.643		7.610		92.393		23.089		39.964		36.929	
Not circumscribed	259.399		7.528		92.513		20.805		40.869		38.324	
Mass internal enhancement		0.120		0.836		0.839		0.201		0.679		0.094
Homogeneous	231.029		6.335		93.677		14.732		38.324		46.973	
Heterogeneous	258.372		7.871		92.168		22.260		41.227		36.510	
Rim enhancement	280.561		6.678		93.366		19.383		40.390		40.195	

IDC = invasive ductal carcinoma, ILC = invasive lobular carcinoma, ER = estrogen receptor, PR = progesterone receptor, HER2 = human epidermal growth factor receptor 2, LVI = Lymphovascular invasion, EIC = Extensive intraductal component

## Discussion

Multivariate survival analysis revealed that patients with breast cancer who showed a higher peak enhancement (DFS hazard ratio, 1.004 [95% CI: 1.001, 1.006]; *P* = .013) on DCE breast MR imaging and triple-negative subtype (DFS hazard ratio, 21.060 [95% CI: 2.675, 165.780]; *P* = .004) had worse DFS. Higher peak enhancement was significantly associated with a higher T stage, higher clinical stage, and higher histological grade. Pathological diagnosis of invasive ductal carcinoma, higher histologic grade, and triple-negative subtype were significantly associated with a higher washout component on the delayed enhancement phase on DCE MR imaging. Our study showed that higher peak enhancement was associated with poorer DFS as well as tumor aggressiveness. In addition, higher washout component was representative of tumor aggressiveness.

Until now, most studies on MR kinetic features using CAD have focused on correlations with histopathologic findings. Leong et al. [18] found that a higher peak signal intensity was seen in ER-negative, PR-negative, and triple-negative tumors, compared to ER-positive, PR-positive, and non-triple-negative tumors. Kinetic features on DCE MR imaging are known to be dependent on the tumor perfusion flow, microvessel density, vascular permeability, and volume of extracellular-extravascular space composition [25–27]. Among CAD-derived kinetic parameters, peak enhancement is considered to reflect the concentration of the contrast agent in both intravascular and extravascular interstitial spaces [8]. In previous reports, high microvessel density and increased vascular permeability were associated with a higher nuclear grade [28], axillary lymph node metastasis, and distant metastasis [4, 21, 29]. Other studies have shown that peak enhancement was significantly greater among patients with lymph node metastasis and lymph node extracapsular extension [30, 31]. We believe that these results might explain the relationship between the peak enhancement on DCE MR imaging and poor prognosis.

Regarding delayed phase kinetic features, a washout kinetic pattern has been reported to correlate with a higher histologic grade, higher Ki-67 expression, increased vascular permeability, and HER2-enriched subtype [18, 32, 33]. In addition, a higher vascular permeability due to increased expression of vascular endothelial growth factor typically found in fast-growing tumors, may cause a higher washout component [8, 34]. Based on these prior results, we expected that a higher washout component on delayed phase enhancement might be associated with a poorer prognosis. In our study, a higher washout component on delayed phase enhancement was associated with the pathological diagnosis of invasive ductal carcinoma, triple-negative subtype, and a higher histologic grade. However, contrary to expectations, there was no statistically significant association between the amount of washout component and survival outcomes. This result was different to that of a recent study; Kim et al. [35] evaluated the association between CAD-generated kinetic features and DFS of patients with primary operable breast cancer, reporting that a higher peak enhancement and washout component were associated with poorer DFS. When comparing with their study, profiles of CAD-generated kinetic features were very different. For example, the mean value of washout component in patients with recurrence was much lower than that of ours (10.38% vs. 39.19%). We speculate that these differences might be caused by using different machines or different protocols. Therefore, using same threshold of CAD-generated features obtained from other institution might be not feasible. Larger studies may be necessary to provide more comprehensive information.

In our study, none of the included morphological findings (shape, margin, internal enhancement) were significantly associated with DFS. Tumors with an irregular shape often occur in high grade breast cancer [36]. However, there was no significant difference in the current study.

In addition to MR imaging, the evaluation of dynamic patterns on breast imaging has also been applied to other modalities. Contrast-enhanced ultrasound (CEUS) imaging has been introduced for the evaluation of tumor kinetics and it enables determinations of the diffusion pattern and real-time quantification of the contrast agent [37–39]. Several quantitative parameters could be automatically calculated on CEUS from the time-intensity curve as follows: peak intensity; time to peak intensity; mean transit time; slope; rise time; and area under the time-intensity curve. CEUS facilitates the functional evaluation of microcirculation and hemodynamic characteristics without extravascular enhancement, which is seen in contrast-enhanced CT and MRI. However, there are no standardized criteria for differentiating between malignant and benign tumors because of different US machines and contrast agents. In addition, there is no general consensus about the methods of imaging acquisition, protocol of contrast agent injection, and ROI setting for time-intensity curve analysis. CEUS is not routinely used in clinical practice because it is difficult to scan an entire breast within a few minutes and time-consuming post-imaging analysis in real-time examinations [40]. Contrast enhanced spectral mammography (CESM) is currently increasing in use, which combines the relative ease, low cost, and practicality of mammography with the high sensitivity of MRI [41–43]. CESM uses intravenous contrast media to obtain the images of enhancing structures. However, dynamic enhancement pattern could not be shown because CESM only shows the existence of enhancement. In the present study, we evaluated the value of CAD-derived kinetic features and our results showed that they might have the potential to be used to characterize tumor aggressiveness and predict the patients’ outcome.

We only included patients who underwent 1.5-T MR imaging because Jansen et al [44] demonstrated that kinetic curves might not be completely consistent across three different MR imaging systems with the same field strength (1.5-T). In another study, qualitative measures of curve shape were not consistent across different field strengths, even when the acquisition parameters were standardized; the maximum percent signal enhancement was significantly higher at 3 T than at 1.5 T [45, 46]. Further studies are required to investigate differences between various MR imagers.

Our study had some limitations. First, this study had a relatively short follow-up period (mean, 55.2 months). It is well known that women with ER-positive tumors remain at risk for late recurrences after primary treatment, which might partly account for the small number of patients with recurrence, making it difficult to observe a robust survival outcome [47]. Second, our results might be difficult to generalize because they were based on a single center, mass lesions, a single imaging protocol, and single CAD system. Third, we could not evaluate inter- or intra- observer agreement of MR kinetic values because the CAD reports were stored prospectively. However, the same results are likely between observers given that we used a standardized ROI policy for choosing image slices that revealed the largest diameter and only mass lesions (non-mass enhancements were excluded). In our experience, significant segmentation differences are markedly decreased in mass lesions compared with non-mass enhancement lesions. In addition, the purpose of our study was not to evaluate the diagnostic accuracy of CAD, but observational survival analysis. Fourth, although these factors might have affected CAD-derived values, we did not consider the effect of the patient’s motion or field inhomogeneity. Finally, of the 32 recurrences, 2 recurrences were detected within the first 6 months from the initial diagnosis, and these could have been due to residual disease.

In conclusion, our findings suggested that preoperative MR imaging kinetic features assessed using a commercially available CAD have the potential to predict survival outcomes. Patients with breast cancer who showed a higher peak enhancement on breast MR imaging may exhibit a worse DFS. In addition, peak enhancement and volumetric analysis of the early and delayed phase enhancements of breast cancers showed correlation with tumor stage, pathological findings, and histological grade, which are indicators of tumor aggressiveness.

## Supporting information

S1 Dataset Detailed characteristics of patients. (XLS)
